# Identification of Fatty Acid Synthase in *Arma chinensis* and Its Expression Profiles in Response to Starvation

**DOI:** 10.3390/insects16020154

**Published:** 2025-02-03

**Authors:** Le Ma, Hongmei Cheng, Changjin Lin, Luyao Fu, Dianyu Liu, Yu Chen, Zhihan Su, Xiaoyu Yan, Wenyan Xu, Xiaolin Dong, Chenxi Liu

**Affiliations:** 1 College of Agriculture, Yangtze University, No. 1 Nanhuan Road, Jingzhou 434025, China; male0402@163.com (L.M.); liudianyu0808@163.com (D.L.); chenyu2632@163.com (Y.C.); suzhihan0505@163.com (Z.S.); yanxiaoyu0316@163.com (X.Y.); 2 Sino-American Biological Control Laboratory, Institute of Plant Protection, Chinese Academy of Agricultural Sciences, No. 2 Yuanmingyuan West Road, Haidian District, Beijing 100193, China; chenghongmei1010@163.com (H.C.); linchangjin163@163.com (C.L.); fuluyao5989@163.com (L.F.); xuwenyan0222@163.com (W.X.)

**Keywords:** fatty acid synthase, starvation, *Arma chinensis*

## Abstract

Fatty acids are the main form of energy production and storage in insects, and fatty acid synthase (FAS) plays a major role in this process. *Arma chinensis* is a natural enemy used in China to control agricultural pests. While its whole genome has been assembled, its FAS genes remain undescribed. Herein, we aim to identify the FAS genes of *A. chinensis* and explore their expression during starvation. Phylogenetic analysis identified nine FAS genes (*ArmaFas1*–*ArmaFas9*). During the predatory developmental stages, *ArmaFas9* expression gradually increased from the 2nd to the 4th instar, peaking at the 5th instar and plateauing at the adult stage. Female adults exhibited the highest expression of *ArmaFas9* in their fat body and the lowest expression in their salivary glands, whereas male adults demonstrated the opposite pattern. *ArmaFas9* expression was negatively correlated with starvation duration. Compared to the controls, starved adult females exhibited a downward expression trend, whereas starved adult males demonstrated an initial increase, followed by a downward trend after 7 days. Starvation and refeeding increased *ArmaFas9* expression. These results demonstrate the important role of *ArmaFas9* in *A. chinensis* growth and development, providing a basis for further studies on lipid synthesis in predatory bugs.

## Simple Summary

Fatty acids are the main form of energy production and storage in insects. Fatty acid synthase (FAS) catalyzes the synthesis of palmitic acid and plays a vital role in lipid metabolism in insects. In this study, we conducted an in-depth analysis of FAS in *Arma chinensis*, a natural enemy used in China for controlling agricultural pests. We investigated the changes in *ArmaFAS9* expression in *A. chinensis* during starvation and starving–refeeding conditions. The outcomes will inform future genetic studies of *A. chinensis* and lay a foundation for FAS gene studies in other insects.

## 1. Introduction

Fatty acids are the main form of energy production and storage in insects. They play a crucial role in key physiological processes such as flight, reproduction, and metamorphosis. Fatty acids are also involved in the growth and development of insects and the synthesis of pheromones [1]. Notably, insects exhibit optimal health when the ratio of saturated fatty acids to monounsaturated fatty acids to polyunsaturated fatty acids in their bodies is 1:1:1. Moreover, lipid reserves provide large long-term energy sources for organisms, playing essential roles in survival and reproduction. In insects, fat accumulation can be stimulated by the digestion of diets rich in sugars and other carbohydrates [2,3]. Lipids play important roles in many physiological processes in insects. For example, many insect species exhibit increased lipid content under conditions of undernutrition, such as before diapause, highlighting the vital role of lipid reserves in their life-history strategies [4,5].

Fatty acid synthase (FAS) is a rate-limiting enzyme that catalyzes the synthesis of palmitic acid, which serves as an energy reserve. Palmitic acid also participates in biological membrane formation, protein acylation reactions, signal transduction, and maintaining the structural integrity of cells [6]. Two types of fatty acid synthases exist in organisms: type I and type II FAS. Type I FAS is present mainly in animals and fungi. Fungal type I FAS is encoded by two genes that assemble into an α6β6 hetero-dodecameric complex [7], whereas mammalian type I FAS is expressed as a single protein and assembles into homodimers [8]. Type II FAS is common in bacterial and eukaryotic organelles, particularly chloroplasts and mitochondria, and is expressed as a discrete protein in the cytosol [9].

In addition to its role in the regeneration of fatty acids, FAS is essential for lipid metabolism. It is a multifunctional enzyme that has been studied to some extent in invertebrates [10]. FASs were first identified in goose liver plasma in 1957 by Wakil et al. [11]. To date, the identification and function of FAS genes have been primarily studied in small insect species [12,13]. The FAS gene was first isolated and purified from the fat body of *Drosophila*, and three distinct FAS genes—*FASCG3523*, *FASCG3524*, and *FASCG7374*—were identified. *FASCG3523* is expressed in fat bodies, whereas *FASCG3524* and FASCG7374 are expressed in oocytes [14]. Notably, in FAS-deficient *Aedes aegypti*, a species of mosquito, triacylglycerol and phospholipid levels are significantly reduced, causing delayed blood digestion and decreased reproductive capacity. This suggests that FAS is required for lipid biosynthesis following blood feeding and that feedback control mechanisms may regulate the rate of fat-body lipid biosynthesis and midgut digestion during feeding [15]. In *Culex pipiens*, another mosquito species, the FAS gene is upregulated during early diapause, which highlights its important role in early fat storage. Moreover, when the expression of the FAS gene is inhibited, females are unable to store the lipids required for overwintering [16]. In *Meteorus pulchricornis*, a parasitoid wasp, the highest expression levels of four FAS genes occur after feeding on a honey diet. Specifically, *MpulFas1* and *MpulFas2* reach peak expression in the adult stage, whereas *MpulFas3* and *MpulFas4* expression peaks in the larval stage [17]. In *Laodelphax striatellus,* a planthopper species, *LsFas1* is expressed throughout all developmental stages, with the highest relative expression observed in the 4th instar and female adult stages. Among different tissues, the highest expression of *LsFas1* is observed in the ovary. Moreover, phylogenetic analysis has revealed that the *LsFas1* cluster is homologous to two FAS of *Nilaparvata lugens* (Brown Planthopper) [18]. Overall, these data highlight the crucial role of FAS in lipid metabolism in insects. Nevertheless, insect FASs have not been extensively studied.

*Arma chinensis*, which belongs to the family Pentatomidae and order Hemiptera, is an excellent predatory natural enemy that preys on many species and can suppress agricultural and forest pests in the orders Lepidoptera and Coleoptera [19,20]. The developmental cycle of *A. chinensis* can be divided into three stages: egg, nymph, and adult. *Arma chinensis* is widely distributed throughout China, mainly in the northeast, north, and other areas [20]. The nutritional utilization from its natural prey and artificial diets has been evaluated using bioassays, nutrigenomics, and metabolomics [21,22,23,24]. In addition, the chemoreception and aggregation-sex pheromones of *A. chinensis* have been functionally characterized and verified [25,26,27]. Notably, *A. chinensis* demonstrates a high tolerance to heat, starvation, and drought, which indicates its ecophysiological adaptation to extreme environmental conditions [28,29,30]. It also exhibits greater resistance to insecticidal pyrethroids than its prey [31], suggesting potential compatibility with chemical insecticides in pest management programs. Moreover, the whole genome of *A. chinensis* has been assembled [32], and the gustatory receptor genes have been identified and classified [26]. However, to the best of our knowledge, its FAS genes have not yet been described.

In the present study, we aim to identify and classify the FAS genes of the whole genome of *A. chinensis* and explore their expression patterns during starvation.

## 2. Materials and Methods

### 2.1. Experimental Insects

In this study, *A. chinensis* insects from a laboratory population, which had been maintained for approximately 70 generations, were used. The insects were fed *Antheraea pernyi* pupae and reared under conditions of 26 ± 1 °C, 65 ± 5% relative humidity, and a 16 h light: 8 h dark photoperiod. Healthy male and female insects with no apparent malformations or nutritional deficiencies were used for bioassays.

### 2.2. Identification of FAS Genes in A. chinensis

To identify members of the FAS gene family in *A. chinensis*, published proteins of *Drosophila melanogaster* were downloaded from the National Center for Biotechnology Information (NCBI) database (Table S1). Subsequently, published proteins were used as query sequences for BLAST 2.11.0+ against a database constructed using the predicted protein sequences of *A. chinensis* (GenBank accession no. PRJNA660864), with a cutoff E-value of <10^−5^. The start and end sites of each gene on the chromosome and the length of each chromosome were obtained from the genome database. A positional map of the gene on the chromosome was generated using TBtools-II (Toolbox for Biologists v1.108). The protein domains of the sequences from the previous step were then queried against the Pfam protein database using HMMER v2.41.2. Motif-based analysis of protein sequences was performed using the Conserved Domains Database online service (https://www.ncbi.nlm.nih.gov, accessed on 15 April 2024). All putative protein sequences from *A. chinensis* were aligned using ClustalW with default parameters. A phylogenetic tree was generated using the MEGAX64 neighbor-joining method, with 1000 bootstrap repetitions.

### 2.3. Prediction of the Three-Dimensional Structure of FAS Protein of A. chinensis

The three-dimensional structure of the target gene was constructed using the homology modeling method. To model FAS1-9, the online prediction software Swiss Model (https://swissmodel.expasy.org/, accessed on 20 May 2024) was used, and modeling targets were selected based on degree of confidence, sequence homogeneity, and query coverage to achieve optimal prediction results. The 3D structure of *Drosophila* FAS was downloaded from the PDB database, and the root-mean-square deviation (RMSD) between the structures was calculated using VMD v1.9.4a53 software.

### 2.4. Analysis of FAS Transcriptome Data of A. chinensis

To quantify the different developmental-stage-specific expression levels of ArmaFas1–9, developmental transcriptome data (entry number PRJNA11234594) were downloaded from the NCBI Sequence Read Archive for eggs, nympha, and adults. For each dataset, low-quality readings were filtered out using Trimmomatic v0.39, and transcript abundance was measured using Kallisto v0.46.2. Data visualization was performed in R 4.3.1 (R Foundation, Vienna, Austria).

### 2.5. Sequence Alignment and Phylogenetic Analysis

The representative FAS protein sequences of *A. aegypti*, *Halyomorpha halys*, *Acyrthosiphon pisum*, *D. melanogaster*, *Bombyx mori*, *Plutella xylostella*, and *Pediculus humanus corporis* were obtained from the NCBI Biotechnology Information database. For the phylogenetic analysis, all putative protein sequences from *A. chinensis,* as well as the FAS gene sequences of the seven insect species, were aligned using the default parameters in ClustalW. A phylogenetic tree was generated using the MEGAX64 maximum-likelihood method, with 1000 bootstrap repetitions.

### 2.6. Quantitative Reverse-Transcription PCR (qRT-PCR)

qRT-PCR was used to validate the expression of FAS genes in *A. chinensis* and the corresponding control groups during different developmental stages, in different organs, under different starvation treatments, and following starvation–refeeding treatments. Total RNA was extracted from all samples using a LanEasy Total RNA kit (LanY Science & Technology Co., Ltd., Beijing, China) according to the manufacturer’s instructions. RNA quality was assessed using a Nano-300 Microspectrophotometer (Hangzhou Allsheng Instruments Co., Ltd., Hangzhou, China), and RNA integrity was confirmed using agarose gel electrophoresis. First-strand cDNA was synthesized using a Fast Quant cDNA kit with a gDNA Eraser (Tiangen Biotech Co., Ltd., Beijing, China) according to the manufacturer’s instructions for qRT-PCR. Primers used to amplify transcripts were based on *ArmaFas9* and designed using Primer 3 v0.4.0 software (https://bioinfo.ut.ee/primer3-0.4.0/, accessed on 10 June 2024) with the following sequences: Forward primer: ^1669^*GCTCGTGGAAAAGCCTCTCT*; reverse primer: ^1952^*AGGCTCCGGTATGACCTTCT*). qRT-PCR was performed using a Quant Studio 5 thermocycler (Applied Biosystems, Foster City, CA, USA) with a PCR reaction volume of 20 μL, containing 2 μL of cDNA, 10 μL of 2 × T5 Fast qPCR Mix (TSINGKE Biotech Co., Ltd., Beijing, China), 0.8 μL of the forward primer, 0.8 μL of the reverse primer, 0.4 μL of 50 × ROX Reference Dye I, and 6 μL of nuclease-free water. The PCR cycling conditions comprised a 95 °C step for 1 min, followed by 40 cycles at 95 °C for 10 s, 60 °C for 5 s, and 72 °C for 10 s. All reactions were run in triplicate, with four independent biological replicates. The dissociation curve was monitored to control for the potential formation of primer dimers. The mRNA expression levels were normalized against *A. chinensis GAPDH* (forward primer: *ACCGTTGAAAAGTGCAAGCC*; reverse primer: *CACAAACGAACATGGGAGCG*) and calculated using the 2^−ΔΔCt^ method [33].

### 2.7. Statistical Analyses

Statistical analyses were performed using GraphPad Prism v8.0.1 (GraphPad Software, San Diego, CA, USA) and SPSS (SPSS 24 software, Chicago, IL, USA). Relative expression levels were analyzed using a one-way analysis of variance with the Waller–Duncan post hoc comparison test. Data are presented as the mean ± standard error of the mean (SEM). Differences were considered statistically significant at *p* < 0.05. Graphs were constructed using GraphPad Prism unless otherwise specified.

## 3. Results

### 3.1. FAS Genes in A. chinensis

**Figure 1:**
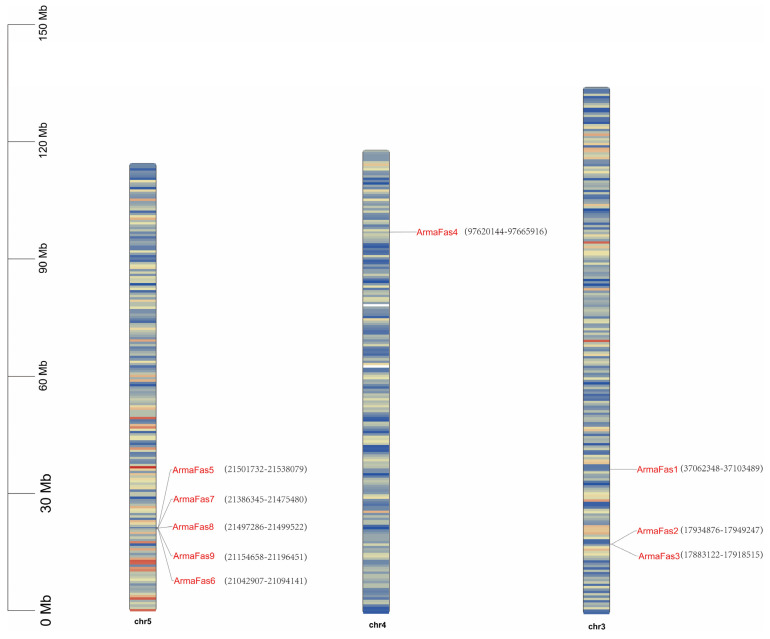
Genome-wide distribution of nine fatty acid synthase genes in *Arma chinensis*. Chromosomal colors, ranging from light to dark, indicate an increase in chromosome density. The numbers represent specific gene loci of the FAS gene.

We identified nine *FAS* genes in the *A. chinensis* genome database and named them *ArmaFas1*–*9*. We mapped the loci of these nine genes to three putative *A. chinensis* chromosomes using chromosomal location analysis (Figure 1). The genes were distributed across different chromosomes rather than being clustered together, and the sizes of the expressed proteins varied, ranging from 751 *(ArmaFas8*) to 3088 (*ArmaFas1*) residues (Table S2). For example, *ArmaFas1*, *ArmaFas2*, and *ArmaFas3* were located on one chromosome, *ArmaFas4* was present on a second chromosome, and *ArmaFas5*, *ArmaFas6*, *ArmaFas7*, *ArmaFas8*, and *ArmaFas9* were closely located on a third chromosome.

**Figure 2:**
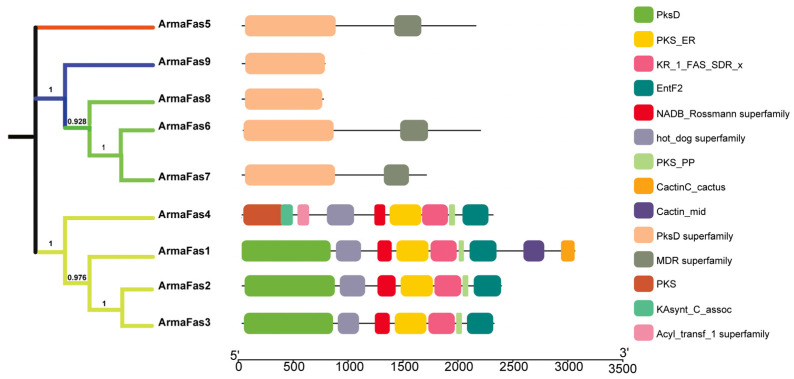
Phylogenetic relationship and domain analysis of the *Arma chinensis* fatty acid synthase genes. Phylogenetic relationship and protein motif analyses of the *Arma chinensis* fatty acid synthase genes. The unrooted phylogenetic tree was constructed using the MEGAX64 neighbor-joining method, and a bootstrap test was performed with 1000 replicates. The colored shadow marks the different ArmaFas families. All motifs were identified via the CDD of the NCBI database using the complete amino acid sequences of ArmaFas. Lengths of motifs for each ArmaFas protein are presented proportionally.

We sequentially compared nine FAS genes of *A. chinensis* (Table S3) and constructed a phylogenetic tree based on 1000 bootstrap repeats using the maximum-likelihood method. Domain analysis indicated that *ArmaFas8* and *ArmaFas9* possess only the PksD superfamily (Figure 2). These results suggest that *ArmaFas1–7* and *ArmaFas8*–*9* do not perform identical functions, possibly due to the distinct roles of the synthesized fatty acids.

**Figure 3:**
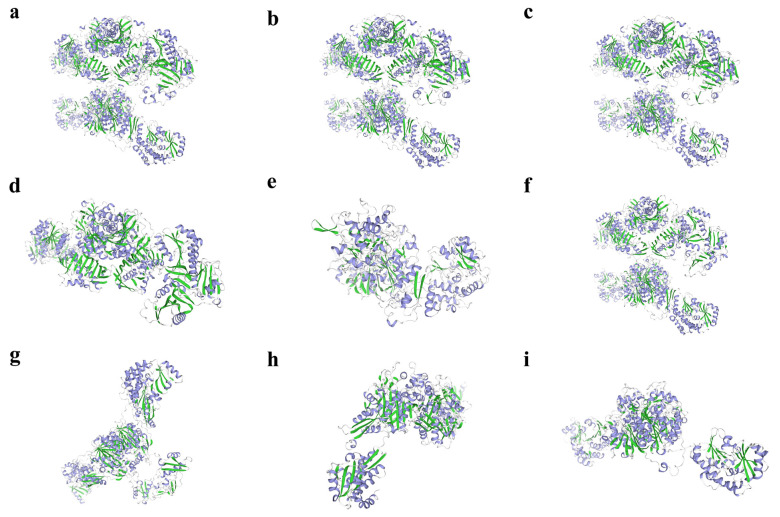
Prediction of the three-dimensional structure of fatty acid synthase proteins of *Arma chinensis*. (**a**–**i**) ArmaFas1–9 proteins, respectively. Green represents the beta fold, and purple represents the alpha helix.

The results of protein tertiary structure prediction revealed the unique three-dimensional conformation of ArmaFas proteins and clearly illustrated its spatial folding pattern (Figure 3). The structure of ArmaFas9 is distinct from that of ArmaFas1–8, with distinct secondary structural elements, such as the number of regular α-helices and β-folds.

**Figure 4:**
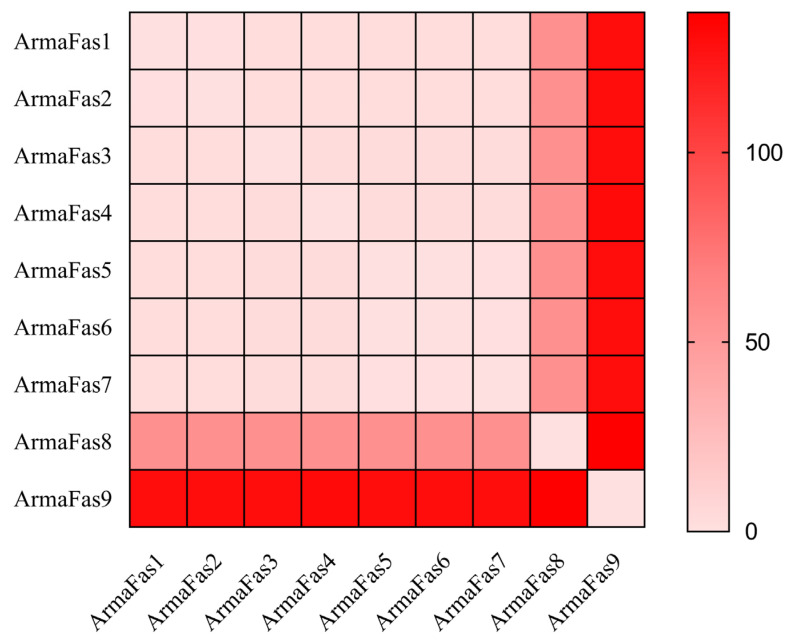
Comparison of the root-mean-square deviation of the predicted structure of ArmaFAS proteins. The brighter the color, the greater the difference in the protein structure.

The RMSDs of the FAS structures of *A. chinensis* were calculated to assess the structural variations (Figure 4). Except for ArmaFAS8 and ArmaFas9, the RMSD between ArmaFAS1–7 were all between 0 and 2. The RMSD of ArmaFas8 was approximately 56, while that of ArmaFas9 was approximately 130. Moreover, the RMSD between ArmaFas8 and the other ArmaFAS proteins was the highest. These results indicate that the 3D structure and conserved motifs predicted for ArmaFas9 were significantly different from those of ArmaFas1–8.

**Figure 5:**
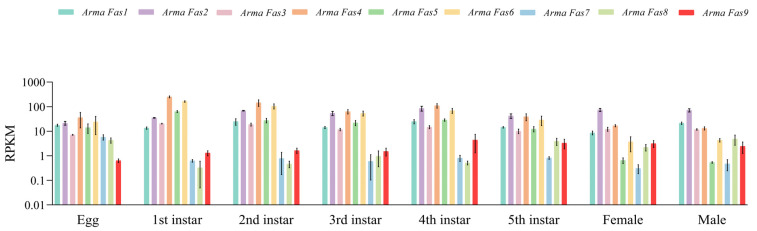
Transcriptomic analysis of *ArmaFas1–9* at different developmental stages. Different colors represent different genes, with the horizontal coordinates indicating the different stages of development and the vertical coordinates representing the relative expression levels.

Transcriptomic sequencing and gene expression analysis of *ArmaFas1–9* revealed that these genes were expressed in different developmental stages of *A. chinensis*, with varying expression levels in each stage (Figure 5). In particular, the expression level of *ArmaFas9* exhibited an increasing trend, ultimately stabilizing in the adult stage.

### 3.2. Phylogenetic Analysis of Insect FAS Genes

**Figure 6:**
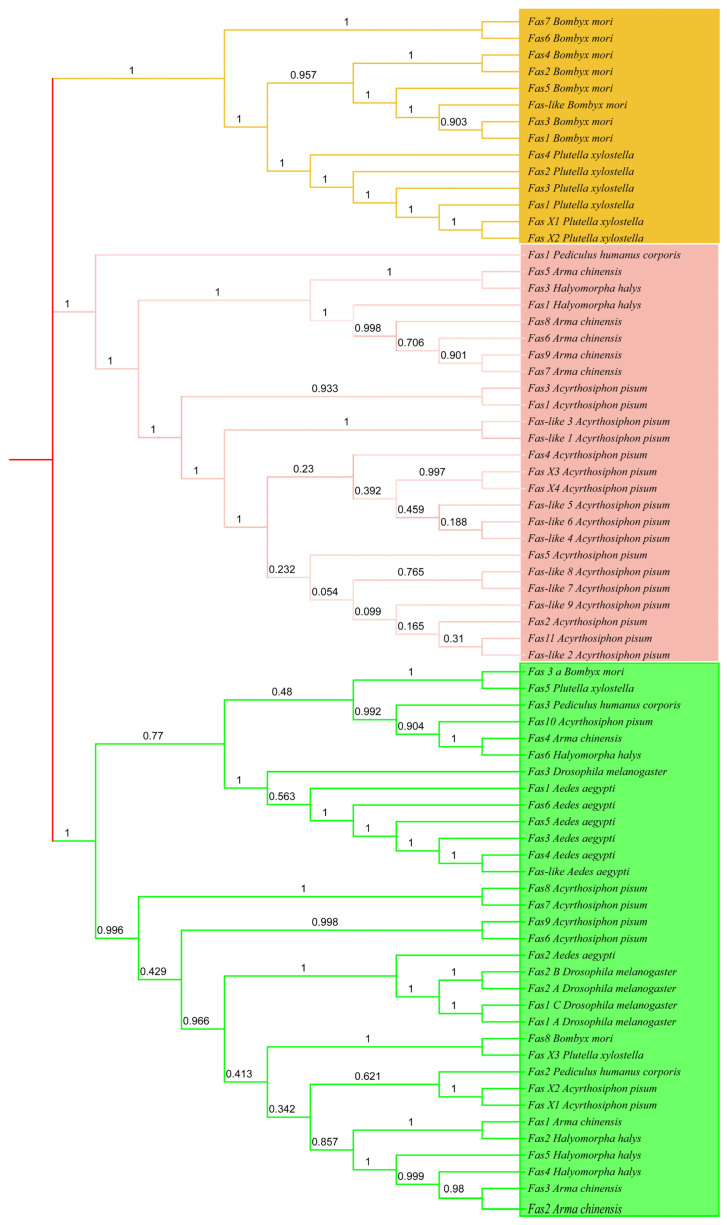
Phylogenetic analysis of *Aedes aegypti*, *Halyomorpha halys*, *Acyrthosiphon pisum*, *Drosophila melanogaster*, *Bombyx mori*, *Plutella xylostella*, *Pediculus humanus corporis*, and *Arma chinensis*. The unrooted phylogenetic tree was constructed using the MEGAX64 maximum-likelihood method, and a bootstrap test was performed with 1000 replicates. Specific colors indicate different branches.

We aligned the amino acid sequences of the nine *A. chinensis* FAS genes with 72 FAS genes from seven other insect species using ClustalW. We also constructed a maximum-likelihood phylogenetic tree containing FAS sequences from all eight insect species (Figure 6). The phylogenetic tree indicated that the FAS sequences of the eight insect species could be divided into three branches, with *A. chinensis* and *H. halys* having the closest evolutionary relationships with FASs.

### 3.3. Tissue, Instar, and Sex-Specific Expression Profiles of ArmaFas9

**Figure 7:**
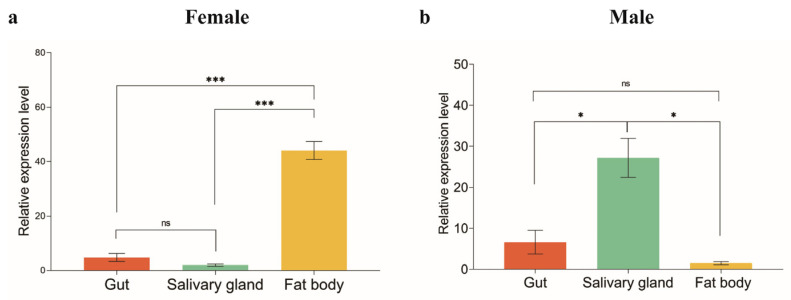
Expression profiles of *ArmaFas9* in different tissues of *Arma chinensis*. (**a**,**b**) qRT-PCR analysis of the expression profiles of *ArmaFas9* in different tissues of female and male *Arma chinensis* adults, respectively. Different tissues are indicated with different colors, and the expression levels are presented on the *y*-axis. Data were analyzed using two-tailed Student’s *t*-tests (n = 3, mean ± SE; ns: *p* > 0.05, * p < 0.05, *** *p* < 0.001). ns: no significance.

As *ArmaFas9* possesses only the PksD superfamily (Figure 2) and exhibits the largest difference in conservative structure (Figure 3 and Figure 4), it may serve as a potential representative for *A. chinensis* FAS genes. Therefore, we determined the expression levels of *ArmaFas9* in the gut, body fat, and salivary glands of male and female *A. chinensis*. We selected 50 *A. chinensis* adults for tissue sampling, with biological replicates performed three times for both males and females. Female adults exhibited the highest level of *ArmaFas9* expression in their fat body and the lowest level in their salivary glands, whereas male adults demonstrated the opposite expression trend (Figure 7a,b).

**Figure 8:**
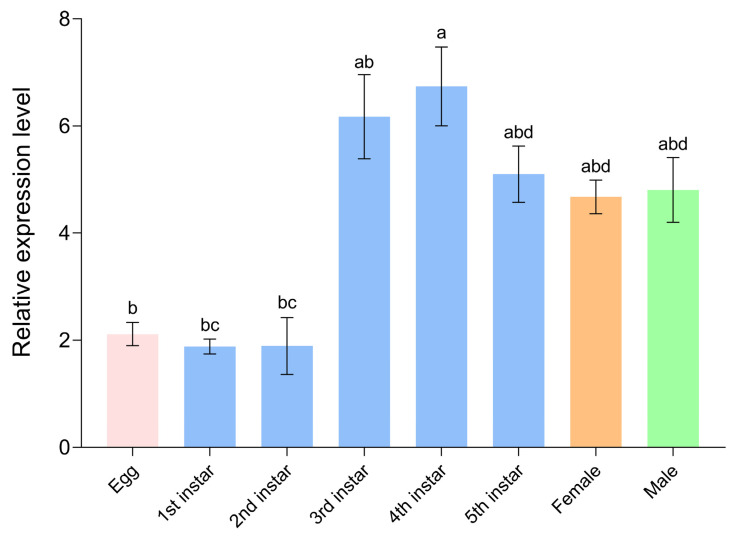
qRT-PCR analysis of *ArmaFas9* expression profiles at different instar nymph stages and adults of *Arma chinensis*. Expression levels are presented on the y-axis. Data represent the mean relative expression levels ± SE. Data were analyzed using one-way analysis of variance (*p*  <  0.05) with the Waller–Duncan post hoc test (n = 3). Different letters on error bars indicate significant differences.

In addition, we measured the expression levels of *ArmaFas9* in eggs, nymphs, adult males, and adult females. We selected five egg masses and 10 *A. chinensis* from nymphs, adult males, and adult females and used three biological replicates. The expression levels of *ArmaFas9* were significantly low in the 1st and 2nd instar, peaking in the 4th instar (Figure 8).

### 3.4. Changes in ArmaFas9 Expression in Response to Starvation Treatments

**Figure 9:**
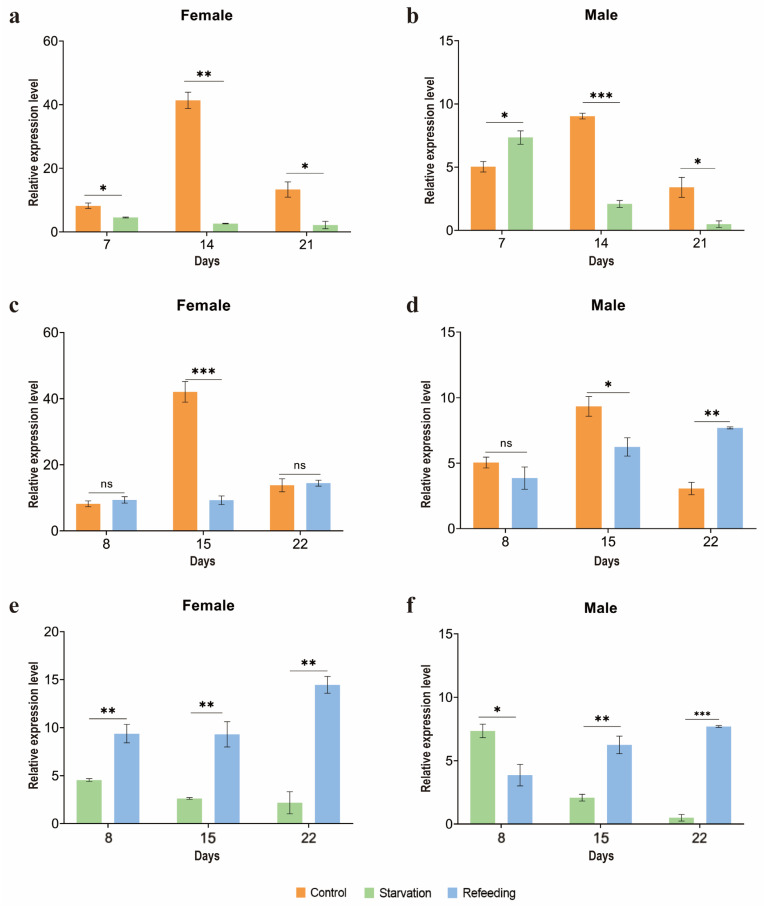
qRT-PCR analysis of the relative expression of *ArmaFas9* under different treatments on different days. (**a**,**b**) Expression of *ArmaFas9* in the starvation and control groups. (**c**,**d**) Expression of *ArmaFas9* in the control and refeeding groups. (**e**,**f**) Expression of *ArmaFas9* in the starvation and repeated refeeding groups. Data are the relative mean expression levels ± SE. Data were analyzed using two-tailed Student’s *t*-tests (n = 3, mean ± SE; ns: *p* > 0.05, * *p* < 0.05, ** *p* < 0.01, *** *p* < 0.001). ns: no significance.

To determine the changes in *ArmaFas9* expression in response to starvation treatments, we starved male and female *A. chinensis* for 7, 14, and 21 days, considering that the longevity of *A. chinensis* adults is approximately 35–40 days. We provided the control group with water. Each treatment was performed using three biological replicates of *A. chinensis*, with each replicate containing ten adults. The results revealed that the expression of *ArmaFas9* was negatively correlated with the number of days of starvation (Figure 9a,b).

Following starvation treatments, the expression of *ArmaFas9* in *A. chinensis* females exhibited a downward trend compared to that in the control group, with significant differences observed on days 7 (*p* = 0.045), 21 (*p* = 0.014), and 14 (*p* = 0.004). In male adults, the relative expression of *ArmaFas9* increased significantly on day 7 (*p* = 0.027) but subsequently decreased significantly as the number of starvation days increased. Specifically, it was significantly decreased on day 14 (*p* = 0.000). In the control groups, the relative expression levels of *ArmaFas9* in both male and female *A. chinensis* initially increased and then decreased. These results indicate that the expression of *ArmaFas9* in *A. chinensis* decreases with increasing starvation time.

### 3.5. Changes in ArmaFas9 Expression in Response to Refeeding Treatments

To determine the changes in *ArmaFas9* expression in response to starving–refeeding treatments, we starved female and male *A. chinensis* adults for 7, 14, and 21 days, followed by feeding for one day. In the control group, male and female adults of the same age were provided with *A. pernyi* pupae. Each treatment was performed using three replicate groups of *A. chinensis*, with each group containing ten adults. The results demonstrated that the expression of *ArmaFas9* was restored after starvation–refeeding (Figure 9c,d).

After starving–refeeding treatment, the expression *ArmaFas9* was significantly different between the experimental and control groups only on day 15 (Figure 9c,d). In female and male adults, there was no significant difference in the relative expression of *ArmaFas9* on day 8 (Figure 9c,d). However, its expression gradually increased in the refeeding group over time, starting from day 15, when it was lower than in the control group (Figure 9c,d), to day 22, when it re-surpassed the control group (Figure 9d). These results indicate that the relative expression of *ArmaFas9* differed between male and female *A. chinensis* following starving–refeeding treatment.

### 3.6. Changes in ArmaFas9 Expression in Response to Starvation and Refeeding Treatments

In female adults, *ArmaFas9* expression in the refeeding group was significantly higher over time than that in the starvation group, with gradually increasing expression levels (Figure 9e). In male adults, the relative expression of *ArmaFas9* in refeeding treatments did not increase significantly on day 8 (Figure 9f) but increased significantly over time, ultimately exceeding the expression levels observed in the starvation treatment groups (Figure 9f). These results indicate that refeeding could restore *ArmaFas9* expression, although this process is delayed in males (Figure 9e,f).

## 4. Discussion

To the best of our knowledge, the present study is the first to describe the FAS genes of *A. chinensis*. Our phylogenetic analysis revealed that *A. chinensis* FAS genes 1–9 could be divided into two major branches, with *FAS1–4* forming one branch and *FAS5–9* forming the other. NCBI (CDD) protein sequence prediction indicated that *ArmaFas1–4* function as a multi-gene family (with more than seven members) but do not contain the PksD superfamily or MDR superfamily. *ArmaFas5–7* are characterized by the presence of the PksD superfamily and MDR superfamily, while *ArmaFas8* and *ArmaFas9* only contain the PksD superfamily. Moreover, using the Swiss Model, we predicted the tertiary domain of ArmaFas1–9 proteins and calculated the RMSD between structures using VMD v1.9.4a53 software. This analysis revealed that the structures of ArmaFas1–4 and ArmaFas6 proteins were considerably similar, whereas those of ArmaFas5 and ArmaFas7–9 proteins were significantly different. This further confirmed that ArmaFas9 exhibited significant structural specificity.

Transcriptome data indicated significant changes in the expression patterns of *ArmaFas1–4* and *ArmaFas5–9*. This distribution may be attributed to FAS, the central enzyme in lipogenesis, which produces a variety of fatty acids and serves as a precursor of other functional lipids [1,34]. Moreover, the FAS genes of *A. chinensis* exhibited the highest similarity to those of *H. halys* and were both located in the same branch of the evolutionary tree. The number of FAS genes in *A. chinensis* is similar to that in *Drosophila* and *H. halys*. Among them, *ArmaFas5–9* were located in the same gene as *H. halys*Fas1 and *H. halys*Fas3, while *ArmaFas1–3* were in the same gene cluster as *H. halys*Fas4 and *H. halys*Fas5. Moreover, *ArmaFas4* was in the same gene cluster as *H. halys*Fas6. Notably, the confidence level for these relationships was between 0.9 and 1, indicating a considerably high similarity. Phylogenetic tree analysis indicated that the similarity of FASs between *B. mori* and *P. xylostella* was the highest (confidence interval [CI], 0.903–1). Most of the FASs of *A. pisum* were similar to those of *ArmaFas5–9* of *A. chinensis*, *H. halys*, and *P. corporis* (CI, 0.054–1). All *A. aegypti* FASs were similar to all *D. melanogaster* FASs, as well as *ArmaFas1–4*, *H. halys*, *P. xylostella*, *P. corporis*, *B. mori,* and some *A. pisum* FASs (CI, 0.342–1). Therefore, it is highly likely that this gene was inherited from a relatively recent common ancestor and has undergone specific changes in subsequent evolution due to factors such as species differentiation and environmental adaptation, resulting in the sequence differences observed today.

The expression pattern of *A. chinensis* FASs in different tissues and developmental stages is consistent with that of *L. striatellus, A. aegypti*, and *Spodoptera litura*, which express FASs throughout all developmental stages and at different ages [6,18,35]. The highest relative expression of *FAS1* in *L. striatellus* is observed during the 4th instar and female adult stages [18]. Among various tissues, *FAS1* expression is the highest in the ovary of *L. striatellus* [18]. In contrast, *A. aegypti* exhibits negligible FAS expression during the larval and pupal stages, whereas in adults, all FAS genes, except *FAS4*, are highly expressed [35]. In *S. litura,* FAS genes are expressed at all ages, with higher expression observed in adult males than in females [6]. In addition, FAS is expressed in the head, midgut, hemolymph, body fat, and epidermis, with the highest expression observed in the body fat of *S. litura* [6]. In the present study, *ArmaFas9* was expressed in *A. chinensis* during all developmental stages. However, its expression in female and male adults differed across various tissues, with opposing patterns observed between the sexes. Specifically, female adults exhibited the highest expression of *ArmaFas* in the fat body and the lowest expression in the salivary glands, whereas male adults exhibited the opposite expression trend. This phenomenon may be explained as follows. In insects, FAS is mainly expressed in body fat and follicle cells, similar to the expression patterns observed in mammalian adipose tissue and the liver [12,36]. This suggests functional differentiation in the expression of FASs [37], potentially attributable to the notable differences in body fat between male and female adults. Body fat is a crucial tissue for maintaining metabolism and reproduction, and it is mainly composed of five cell types that vary in composition, size, and function across different developmental stages [38]. Trophoblasts are pleomorphic cells that originate from the mesoderm and are involved in the storage, secretion, and detoxification of organic materials in the insect body [39]. Female insects generally have a higher number of trophoblast cells than males but lower production of vitellogenin [40]. This further supports the notion that differences in the fat body between adult *A. chinensis* females and males contribute to the functional differentiation of FAS.

In the present study, the expression of *A. chinensis* FAS decreased under starvation conditions. This is because starvation reduces the substrates available to *A. chinensis* for obtaining food and energy from the environment, resulting in decreased *ArmaFas9* expression. Notably, we observed that *ArmaFas9* expression in starved adult males first increased and then decreased, whereas that in starved adult females decreased continuously. In addition, *ArmaFas9* expression was higher in the starvation–refeeding group than in the control group, likely because under the pressure of mild starvation, *A. chinensis* could utilize other sugars to maintain blood glucose and trehalose homeostasis to sustain basic energy metabolism [41]. However, as the severity of starvation increases, lipids, particularly triglycerides, are broken down to increase the resistance to starvation, eventually leading to autophagy. As nutrient-rich conditions are uncommon in natural environments, most organisms must endure repeated episodes of starvation. To maintain the minimum levels of energy and essential components required for survival, organisms have evolved mechanisms for digesting their cellular components. One such mechanism is autophagy, which is now recognized as a critical cellular system for survival during nutritional stress. During starvation, some cells undergo autophagy to generate nutritional resources that support the survival of other cells [42]. Autophagy targets and degrades different types of nutrient stores, generating various metabolites and fuels, including amino acids, nucleotides, lipids, and carbohydrates [43]. As female adults have more fat storage than male adults, we postulate that they also have a stronger tolerance for hunger than male adults. Therefore, male adults may enter autophagy earlier, after 8 days of starvation. This may explain our observation of the significantly higher *ArmaFas9* in the male starvation group than in the refeeding group (Figure 9f). In contrast, *ArmaFas9* expression in female adults in the starvation group was significantly lower than that in the refeeding group (Figure 9e). This may explain the increased expression of FAS in *A. chinensis* in response to starvation. Specifically, as FAS is a rate-limiting enzyme in *A. chinensis*, when food became available again, its expression in *A. chinensis* increased and further increased under the influence of autophagy.

## 5. Conclusions

In the present study, we conducted an in-depth analysis of FAS 9 in *A. chinensis*. Notably, this study is the first to describe FAS genes in *A. chinensis*. Specifically, we investigated the changes in *ArmaFAS9* expression in *A. chinensis* during starvation and starving–refeeding conditions. The results of this study will inform future genetic studies of *A. chinensis* and lay a foundation for FAS gene studies in other insects.

## Data Availability

Data will be made available upon reasonable request from the corresponding author.

## Supplementary Materials

The following supporting information can be downloaded at: https://www.mdpi.com/article/10.3390/insects16020154/s1, Table S1: Query sequences of the published proteins of *Drosophila melanogaster*; Table S2: Number of amino acid residues in *A. chinensis* fatty acid synthase; Table S3: Multiple sequence alignment of *A. chinensis* FAS.

## Abbreviations

The following abbreviations are used in this manuscript:

CI	Confidence interval
FAS	Fatty acid synthase
NCBI	National Center for Biotechnology Information
RMSD	Root-mean-square deviation
SEM	Standard error of the mean
qRT-PCR	Quantitative Reverse-Transcription PCR
